# Electrocatalytic performances of g-C_3_N_4_-LaNiO_3_ composite as bi-functional catalysts for lithium-oxygen batteries

**DOI:** 10.1038/srep24314

**Published:** 2016-04-14

**Authors:** Yixin Wu, Taohuan Wang, Yidie Zhang, Sen Xin, Xiaojun He, Dawei Zhang, Jianglan Shui

**Affiliations:** 1 School of Chemistry and Chemical Engineering, Hefei University of Technology, Hefei, 230009, China; 2 School of Chemistry and Chemical Engineering, Anhui University of Technology, Maanshan, 243002, China; 3 CAS Key Laboratory of Materials for Energy Conversion, Hefei, 230026, China; 4 School of Materials Science and Engineering, Beihang University, Beijing, 100191, China

## Abstract

A low cost and non-precious metal composite material g-C_3_N_4_-LaNiO_3_ (CNL) was synthesized as a bifunctional electrocatalyst for the air electrode of lithium-oxygen (Li-O_2_) batteries. The composition strategy changed the electron structure of LaNiO_3_ and g-C_3_N_4_, ensures high Ni^3+^/Ni^2+^ ratio and more absorbed hydroxyl on the surface of CNL that can promote the oxygen reduction reaction (ORR) and oxygen evolution reaction (OER). The composite catalyst presents higher activities than the individual components g-C_3_N_4_ and LaNiO_3_ for both ORR and OER. In non-aqueous Li-O_2_ batteries, CNL shows higher capacity, lower overpotentials and better cycling stability than XC-72 carbon and LaNiO_3_ catalysts. Our results suggest that CNL composite is a promising cathode catalyst for Li-O_2_ batteries.

Li-O_2_ battery is a promising energy storage solution, as it has a theoretical specific energy of 5200 Wh kg^−1^ (including O_2_), much higher than the state-of-the-art Li-ion battery^1^,^2^,^3^. A typical non-aqueous Li-O_2_ cell is composed of a piece of lithium metal as the anode, electrolyte and a piece of porous air electrode as the cathode. During the discharge reaction, the oxygen reduction reaction happens in the cathode producing lithium peroxides (Li_2_O_2_)^4^. During the charge, a reversal reaction of oxygen evolution reaction happens, decomposing Li_2_O_2_ to oxygen and Li-ions again^5^. It is crucial to utilize highly efficient catalysts to promote the ORR/OER process. So far, many metals, metal oxides, carbonaceous materials and redox media have been reported as cathode catalysts in Li-O_2_ batteries^6^,^7^,^8^,^9^,^10^. The noble metal and noble metal oxide nanoparticles are usually the highly efficient catalyst, however, the high cost of the noble metals have limited their large-scale applications^11^,^12^.

Graphitic carbon nitride (g-C_3_N_4_) polymer, has a planar phase analogous to graphite. Unlike graphite, however, g-C_3_N_4_ has both 3-fold-coordinated (graphite-like) and 2-fold-coordinated (pyridine-like) nitrogen atoms, and every carbon atom is bonded to three nitrogen atoms, including both pyridinic and graphitic nitrogen moieties^13^. g-C_3_N_4_ is an attractive catalysis material due to its properties like high chemical stability, low-cost preparation and specificity in the structure and composition^14^. So far, it has been used in many areas, such as the photochemical splitting of water, fuel cells, and metal-free heterogeneous catalysis of various organic reactions^15^,^16^,^17^,^18^,^19^,^20^. In particular, g-C_3_N_4_ contains alleged “nitrogen pots” with six nitrogen lone-pair electrons, which enable the material to modify the electronic structures of the molecular and to be ideal sites for metal inclusion^21^,^22^. However, the poor electroconductivity and the relatively low ORR catalytic activity limit the electrocatalytic application of g-C_3_N_4_^23^. These problems can be solved by composting g-C_3_N_4_ with other materials which have better electrical conductivity. For example, g-C_3_N_4_ combined with non-noble metals like Co-g-C_3_N_4_^24^, Fe-g-C_3_N_4_^25^, and Ni-Co_3_O_4_-C_3_N_4_^26^ have been investigated as the effective catalysts in fuel cells and Li-O_2_ batteries. The composites have shown promising ORR and OER activity and tolerance ability that are comparable to commercial Pt/C.

Perovskite oxides (ABO_3_) have been widely used as catalysts for fuel cells and metal-O_2_ batteries^27^, owing to their unique structures, excellent oxygen mobility and low cost. LaNiO_3_ has the intrinsic activity for both ORR and OER among the perovskite type oxides, because Ni^3+^ at B site in the perovskite structure has single electron-filled e_g_ orbit, and therefore, provides the favorable bond energy of M (B site)-O bond for ORR and OER^28^. In addition, the catalytic activity of LaNiO_3_ could be readily enhanced through increasing the Ni^3+^/Ni^2+^ ratio by replacing part of Ni ions with Mg or Fe ions. g-C_3_N_4_ has a large number of removable electronic between layers, and cavities encircling with some pyridine-like nitrogen^29^. So it has a high ability to complex with abundant metal ions and adjust its conjugation structure subsequently. Therefore combining g-C_3_N_4_ with LaNiO_3_ can theoretically improve the electron transfer and catalytic activity of the CNL composite. However, to the best of our knowledge, g-C_3_N_4_ have never been composited with LaNiO_3_ to explore the enhanced the catalytic activity for both ORR and OER.

In this work, we synthesized a composite catalyst g-C_3_N_4_-LaNiO_3_ (CNL), and demonstrated that the CNL has significantly enhanced electrocatalytic activity for OER and ORR compared with individual component g-C_3_N_4_ or LaNiO_3_ in an alkaline electrolyte. Furthermore, CNL exhibited improved round-trip efficiency and cycling stability in Li-O_2_ batteries. These results suggest that the CNL is a promising bi-functional catalyst for oxygen oxidation/evolution reactions.

## Results and Discussion

### Characterization of CNL catalyst

**Figure 1:**
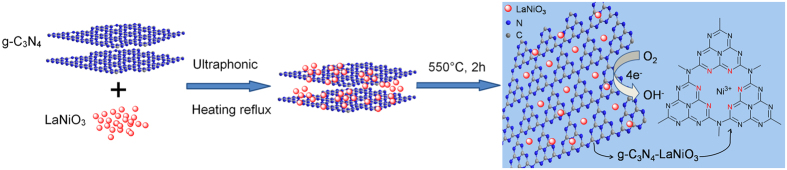
Schematic illustration of the synthetic process of CNL composite.

**Figure 2:**
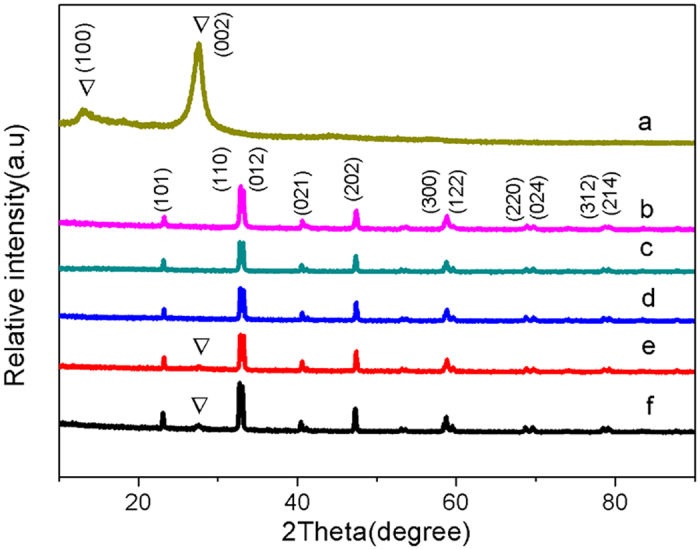
XRD patterns of (**a**) g-C_3_N_4_, (**b**) LaNiO_3_, (**c**) 5 wt.% CNL, (**d**) 10 wt.% CNL, (**e**) 20 wt.% CNL, (**f**) 30 wt.% CNL hybrid catalyst. The weight percentage number imply the g-C_3_N_4_.

Figure 1 depicts the synthetic process of CNL catalyst and the molecular structure of the catalytic spots of CNL catalyst. Figure 2 displays the XRD patterns of pure g-C_3_N_4_, LaNiO_3_ and the hybrid catalyst CNL with different weight percent of g-C_3_N_4_. It can be seen that the pure g-C_3_N_4_ has two distinct peaks. The strong peak at 27.39°, corresponding to the (002) peak with in-planar distance of 0.326 nm (JCPDS 87–1526), is attributed to the long-range in-planar stacking of aromatic units^30^. Another peak at 13.08°, with a much weaker intensity, can be assigned to (100) diffraction peak^20^, which corresponds to a distance of 0.686 nm, belongs to tri-s-triazine units, due to the interlayer stacking. After the LaNiO_3_ being composited with g-C_3_N_4_, the CNL hybrids show a coexistence phases of both g-C_3_N_4_ and LaNiO_3_, although the characteristic peak of g-C_3_N_4_ was not obvious for the 5 wt.% and 10 wt.% CNL hybrids.

**Figure 3:**
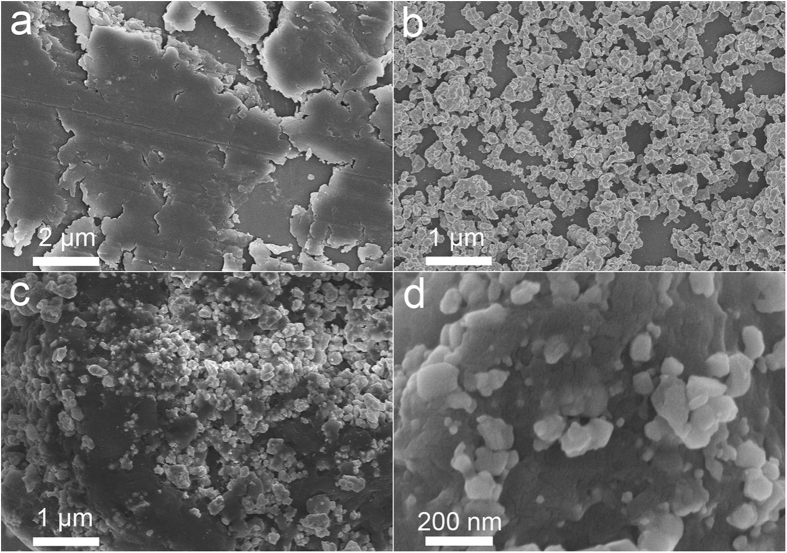
FE-SEM images of (**a**) g-C_3_N_4_, (**b**) LaNiO_3_, (**c**) 10 wt.% CNL and (**d**) magnified images of 10 wt.% CNL hybrid catalyst.

For the further information of the materials microstructure, Fig. 3 shows the SEM images of g-C_3_N_4_, LaNiO_3_ and 10 wt.% CNL. Pure g-C_3_N_4_ shows an aggregated layered structure with smooth surface. The agglomerate size is several micron meters. LaNiO_3_ particles are hundreds nanometers large, more than one order of magnitude smaller than g-C_3_N_4_. As to the image of 10 wt.% CNL, LaNiO_3_ particles are deposited on the surface of g-C_3_N_4_.

**Figure 4:**
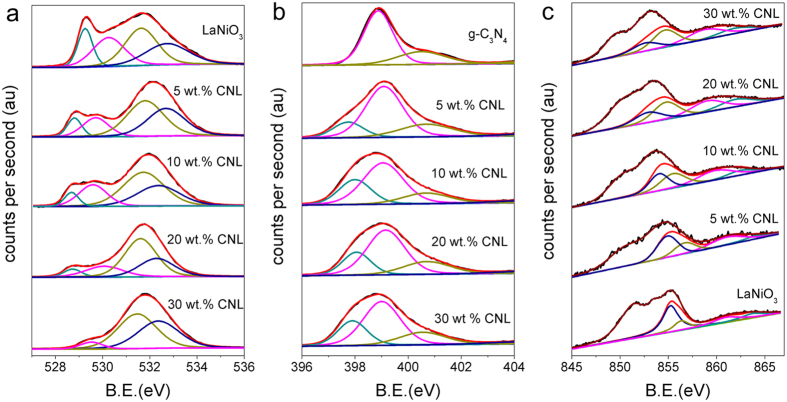
(**a**) O 1s XPS spectra, (**b**) N 1s XPS spectra and (**c**) Ni 2p^3/2^ XPS spectra of LaNiO_3_ and x wt.% CNL (x = 5, 10, 20 and 30) composite catalysts.

In this study we used the X-ray photoelectron spectra to research the physical and chemical characteristic of the composite catalyst surface. Figure 4a shows the O 1s XP-spectra of g-C_3_N_4_, LaNiO_3_ and CNL catalysts. The O 1s XP-spectra contains four distinct species. Two low binding energy peaks located at 528.6 eV and 530.1 eV are ascribed to the lattice oxygen, O^2−^ species in lanthanum oxide and nickel oxide^31^. The peaks located at 531.5 eV and 532.2 eV are from lanthanum and nickel hydroxides^32^,^33^. With the increase of g-C_3_N_4_, the peaks at low binding energy become weaken, and the peaks at high binding energy become stronger, indicating that the g-C_3_N_4_ compositing LaNiO_3_ can promote the adsorption of hydroxyl on the surface of the composite catalyst and the covalency of Ni-O bond become stronger^34^. It has been demonstrated that the rate of O_2_^2−^/OH^−^ displacement and OH^−^ regeneration dominate the ORR kinetics, and the great covalence of the Ni-O bond can promote the ORR kinetics^30^. Furthermore, OER is either through chemisorbed OOH^−^ formation or oxidation of surface OH^−^. The rate-determining step of the OER on perovskite oxides is governed by the concentration of hydroxide species that participate in the formation of the O-O bond in hydroperoxide. Thus, the high lattice hydroxide concentration on the surface can promote the HOO^−^ forms, and consequently has a positive effect on OER activity.

The N 1s XPS spectrum of the g-C_3_N_4_ shown in Fig. 4b can be fitted into three peaks at 398.8, 400.5 and 404.7 eV, corresponding to pyridine-like N, pyrrole-like N, and oxidized N, respectively^35^,^36^. After adding g-C_3_N_4_, a new peak emerged at 398.2 eV, which also corresponds to pyridine-like N. The reason why it was lower than 398.8 eV, can be explained by the existence of chemical bonds in the g-C_3_N_4_-LaNiO_3_ heterojunction.

Ni 2p^3/2^ was deconvoluted into two peaks. The binding energy of 854.3 eV was due to Ni^2+^, and 856 eV was due to Ni^3+^ as shown in Fig. 4c 31. The peak intensity of the lower oxidation state (Ni^2+^) is weakening and the higher oxidation state (Ni^3+^) is enhancing with the increase of g-C_3_N_4_ in the composite. It suggests that through heat treating LaNiO_3_ could be doped with nitrogen-atoms forming LaNiO_3−x_N_x_, which promoted the formation of Ni^3+^ on the surface of the composite catalyst. According to previous report, the existence of Ni^3+^ in LaNiO_3_ are beneficial to both ORR and OER activities of the material^37^.

### Electrocatalytic performance

**Figure 5:**
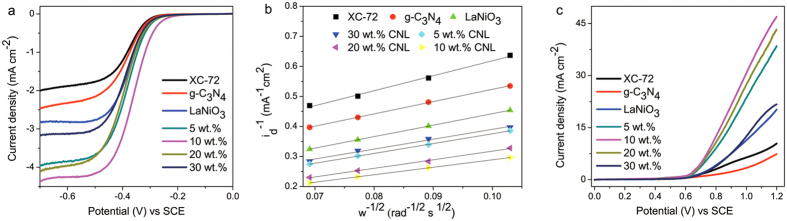
(**a**) ORR polarization curves, (**b**) Koutecky-Levich plots and (**c**) OER polarization curves of XC-72, g-C_3_N_4_, LaNiO_3_ and x wt.% CNL (x = 5, 10, 20 and 30) catalysts.

The typical polarization curves for the ORR was investigated in O_2_ saturated 0.1 M KOH solution, shown in Fig. 5a. The ORR onset potential of 10 wt.% CNL is about −0.286 V, the most positive among that of LaNiO_3_ (−0.304 V), 20 wt.% CNL (−0.319 V), 30 wt.% CNL (−0.315 V), g-C_3_N_4_ (−0.315 V) and XC-72 (−0.314 V). And the half-wave potential of 10 wt.% CNL is −0.35 V, ~350 mV higher than the 2^nd^ high value of 5 wt.% CNL. In additional that the 10 wt.% CNL also exhibits the largest diffusion-limiting current, and the limiting current was 4.36 mA cm^−2^, much higher than 2.82 mA cm^−2^ of LaNiO_3_. Furthermore, for kinetic analysis the current-potential curves at various rotation speeds were also analyzed with the Koutecky-Levich equation:


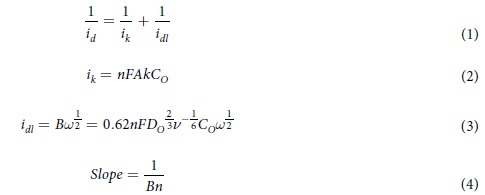


This equation has been widely used to analyze the ORR reaction kinetics^38^,^39^,^40^, where *i*_*d*_ is the tested disk currentdensity, *n* is the number of electron transfer in the ORR, *k* is the Boltzmann constant, *F* is the Faraday constant, *A* is the geometric area of the disk electrode, *C*_*O*_ is the saturated concentration of oxygen in 0.1 M KOH solution, *D*_*O*_ is the diffusion coefficient of oxygen, *ν* is the kinematic viscosity of the 0.1 M KOH solution and ω is the electrode rotation rate. There should be a linear relationship between *i*_*d*_^−1^ and *ω*^−1/2^, the intercept is equal to *i*_*k*_^−1^, and *n* could be calculated from the slope.

Figure 5b shows the Koutecky-Levich plots of XC-72, g-C_3_N_4_ and CNLs based on ORR polarization curves at −0.6 V with rotation speed of 2000, 1600, 1200 and 900 rpm. All curves exhibit good linear characteristic, indicating the first-order kinetics characteristics of the ORR. According to equations (3 and 4), the *n* value could be calculated as shown in Table 1. The *n* value of 10 wt.% CNL is about 3.86, indicating the ORR was a four-electrons process. In comparison with LaNiO_3_ catalyst, as-synthesized 10 wt.% CNL material delivers a better ORR activity and kinetics. The higher electrocatalytic activity is due to the high Ni^3+^/Ni^2+^ ratio in 10 wt.% CNL, which could promote adsorption/desorption of oxygen and thus the ORR activity in an alkaline electrolyte. Therefore in this work, the composite strategy has demonstrated to be highly effective to improve the ORR catalytic activity of the components.

The catalytic activity of the synthesized CNL for OER was also investigated by the RDE technique by measuring the polarization curves in O_2_ saturated 0.1 M KOH solution from 0.0 V to 1.2 V, at a scan rate of 5 mV s^−1^ and a rotation speed of 1600 rpm. As shown in Fig. 5c, all the synthesized composite catalysts exhibit much better catalytic activity for OER than XC-72 carbon and LaNiO_3_. The OER onset potential for LaNiO_3_ is 0.70 V and the OER diffusion-limiting current density at 1.2 V is 20.2 mA cm^−2^. For a comparison, the 10 wt.% CNL has a more negative onset potential of 0.63 V, and the current density is 47.0 mA cm^−2^ at 1.2 V remarkably higher than that of LaNiO_3_.

This improved property of 10 wt.% CNL is mainly due to the enhanced adsorption of hydroxyl on the surface of CNL composite. The hydroxyl participates in the formation of HOO^−^, and the increase of the HOO^−^ concentration leads to the high OER electrocatalytic activity. As to the elexctrocatalytic activity of g-C_3_N_4_, due to the stronger electro-negativity of N than C, the electron will transfer from C to adjacent N, while the π-conjugation causes the electrons to return to the adjacent Cp^2^ orbital^41^,^42^. These donation and back-donation processes not only help the O^2−^ adsorption by the strong chemical bond between O and C, but also promote O_2_ desorption on the adjacent C atoms, resulting in enhanced ORR and OER activity. The reason that ORR and OER activities of the 20 wt.% CNL and 30 wt.% CNL are lower than these of 10 wt.% CNL, can be interpreted by the worsen electron conductive environment due to too much non-conductive in the composites.

**Figure 6:**
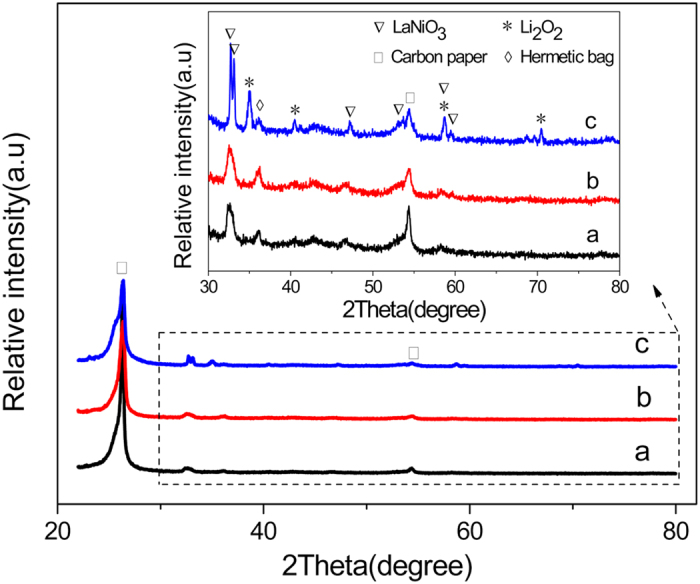
XRD patterns of the 10 wt.% CNL cathode for Li-O_2_ batteries at (**a**) pristine, (**b**) 1st charged and (**c**) 1st discharged condition (In order to prevent the Li_2_O_2_ decomposition under the influence of water in the air, so put the cathode in a hermetic bag for XRD testing).

XRD was used to confirm the formation and decomposition of Li_2_O_2_ on the 10 wt.% CNL cathode during the charge-discharge process in non-aqueous Li-O_2_ batteries. Figure 6 shows XRD patterns of the 10 wt.% CNL cathode at pristine,1st discharge and 1st recharge condition. After 1st discharge, compared to the XRD pattern of the pristine cathode, some diffraction peaks at 34.7, 40.3, 58.3 and 70.5° appear, which can be ascribed to Li_2_O_2_ formation during the discharging process. After the 1st charge process, the diffraction peaks of Li_2_O_2_ disappear, indicating that the formed Li_2_O_2_ in the 1st discharge is decomposed during the charging process. This XRD pattern evolution demonstrates the rechargeability of this 10 wt.% CNL cathode.

**Figure 7:**
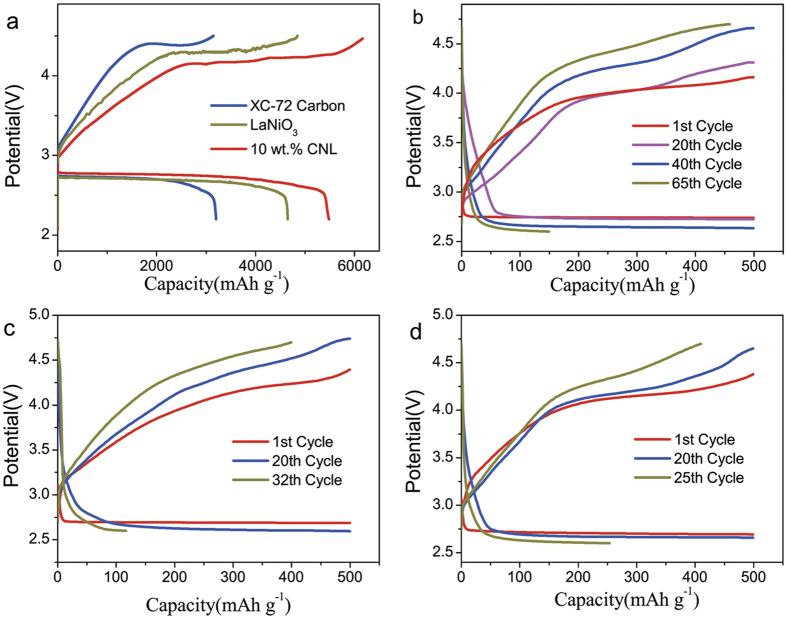
Electrochemical performances of Li-air batteries. (**a**) The first charge/discharge curve of Li-air batteries using XC-72 carbon, LaNiO_3_ and 10 wt.% CNL as the air electrode at a current density of 50 mA g^−1^. (**b**–**d**) Discharge and charge curve of the cells at a current density of 250 mA g^−1^ using 10 wt.% CNL, LaNiO_3_ and XC-72 carbon as the air electrode, respectively.

We further investigated the catalyst activity of the composites in non-aqueous Li-O_2_ batteries. The first discharge and charge profiles of XC-72, LaNiO_3_ and 10 wt.% CNL in the Li-air batteries were compared at a current density of 50 mA g^−1^ (Fig. 7a). In particular, the initial discharge capacity of the 10 wt.% CNL is 5500 mAh g^−1^, higher than that of LaNiO_3_ (4600 mAh g^−1^) and XC-72 carbon (3600 mAh g^−1^). Also, it can be clearly seen that the discharge voltage plateau of the 10 wt.% CNL catalyst was ~2.8 V, higher than that of the LaNiO_3_ (~2.7 V) and the XC-72 carbon (~2.7 V). The charge voltage plateau of the 10 wt.% CNL was ~4.0 V, lower than that of LaNiO_3_ (~4.3 V) and XC-72 carbon (~4.4 V). The smaller overvoltages of CNL suggests the higher catalytic activity than LaNiO_3_ and XC-72. In additional, 10 wt.% CNL catalyst presented 65 cycles between voltage window of 2.6 V to 4.7 V as shown in Fig. 7b. While the performance of the LaNiO_3_ and XC-72 carbon catalyst are only up to 32 and 25 cycles respectively. These results indicate that the 10 wt.% CNL as air electrode catalyst enhanced the capacity and cyclability of Li-O_2_ batteries.

In conclusion, LaNiO_3_ and g-C_3_N_4_ composite was made as the bi-functional catalyst for oxygen reactions. The composite material delivers batter ORR and OER activities than individual LaNiO_3_ and g-C_3_N_4_, the reason is that the CNL catalyst has a high Ni^3+^/Ni^2+^ ratio that can promote adsorption/desorption of oxygen in an alkaline electrolyte, and more absorbed hydroxyl on the surface of CNL that can promote HOO^−^ formation. LaNiO_3_ and g-C_3_N_4_ composite is for the first time used as the bi-functional catalysts for lithium air batteries. The composite presents enhanced specific capacity and cycle stability. The results suggest CNL composite can be a potential bifunctional catalyst for the Li-O_2_ batteries, and the composite strategy is worth of further investigation.

## Methods

### Synthesis of LaNiO_3_ NPs and g-C_3_N_4_ sheets

LaNiO_3_ nanoparticles (NPs) were synthesized by a sol-gel method. 1.3 g La_2_O_3_ (Sinopharm, reagent grade) was dissolved in 10 mL HNO_3_ solution (65% ≤ HNO_3_ ≤ 68%), obtaining the La(NO_3_)_3_ · xH_2_O solution. Then 2.37 g Ni(NO_3_)_2_ · 6H_2_O (Sinopharm, reagent grade) and the La(NO_3_)_3_ · xH_2_O were dissolved in 100 ml de-ion water. Subsequently, the mixture of 6.75 g citric acid (HOC(COOH)(CH_2_COOH)_2_ ≥ 99.5%) and 3.6 mL ethylene glycol (HOCH_2_-CH_2_OH, 99.8%) were dropwise added into the metal nitrates under magnetic stirring at 80 °C. The molar ratios of total metal cations: citric acid: ethylene glycol were 1:2:4. Then the resulting solution was stirred at 80 °C to evaporate water forming a viscous gel. The gel was heated at 400 °C for 1 hour, and the amorphous citrate precursor was obtained. The precursor was milled and then calcinated in oxygen atmosphere at 750 °C for 5 hours to obtain final LaNiO_3_ powders.

Bulk g-C_3_N_4_ sheets were synthesized by pyrolysis method. Briefly, 5 g melamine were heated at 550 °C for 2 hours with a heating rate of 5 °C min^−1^. The resultant agglomerates were ground for 1 hour in a mortar, then further heated at 550 °C for 2 hours. Then the as-prepared bulk g-C_3_N_4_ were obtained.

### Synthesis of CNL composite material

The CNL were synthesized as follows: 0.04 g g-C_3_N_4_ were dispersed in 50 mL ethanol in a beaker, and treated by ultrasonic for 30 min to fully disperse g-C_3_N_4_. Then 0.4 g LaNiO_3_ NPs were added into the g-C_3_N_4_ suspension, and the mixture were further dispersed by the ultrasonic treatment for 30 min, and then heated under reflux for 6 hours at 90 °C with stirring. Then evaporated the alcohol completely with stirring, a precipitate was obtained. The precipitate was collected and heated at 550 °C for 2 hours in nitrogen atmosphere. Then the resultant compound were ground for 1 hour in the mortar, to make the final 10 wt.% CNL composite material (the weight percentage number imply the g-C_3_N_4_). By this method, CNL catalysts in different mass ratios (5 wt.% CNL, 20 wt.% CNL and 30 wt.% CNL) were also prepared.

### Characterization of materials

The crystal phase and purity of the as-prepared powders were determined by X-ray powder diffraction with Cu Kα radiation (λ = 0.154056 nm) from 10° to 90°. The structures of the samples were determined by field-emission scanning electron microscope (SEM) performed on a Hitachi SU8020 at an acceleration voltage of 3 kV. X-ray photoelectron spectra (XPS) data were obtained on a Rigaku D/MAX2500V X-ray photoelectron spectrometer with an exciting source of Al Kα (1486.6 eV).

### Electrochemical measurements

Electrochemical characterizations of the samples were performed on a rotating-disk electrode (RDE, ATA-1B) with a three-electrode cell. A platinum electrode was used as the counter electrode. A saturated calomel electrode was used as the reference electrode. A glassy carbon electrode was used as the working electrode. 0.1 M potassium hydroxide (KOH) solution was used as the electrolyte. For CV, ORR and OER studies, the 1 mg composite catalyst powder was mixed with 2.5 mg XC-72 carbon to ensure sufficient electronic conductivity. The as-prepared catalyst and 30 μL of 5 wt.% Nafion solutions were dispersed in 1 mL de-ion water/isopropanol solution (the volume ratio of de-ion water: isopropanol is 5:1), with an ultrasonic treatment for 40 min to make a homogeneous ink. The glassy carbon electrodes (3 mm diameter, 0.071 cm^2^ area) were polished on a clean polishing cloth with the 0.05 μm alumina slurry, rinsed and dried. Then we pipeted 2.8 μL of the suspension drop-cast and loaded it on the glassy carbon, which was dried slowly inside a closed container. The glassy carbon electrode loaded with catalyst was immersed into a N_2_-saturated KOH electrolyte for the steady-state CVs. The N_2_ was switched to O_2_ and purged for another 30 min. After that the ORR polarization curve was measured from 0 V to −1.0 V. Then, switched the O_2_ to N_2_ and purged for 30 min. Subsequently, the OER polarization curve were examined by a voltage scan from 0.0 V to 1.2 V. Electrochemical data were collected with an Autolab electrochemical workstation.

For Li-O_2_ battery studies, the battery were fabricated in a glove box which was filled with pure argon gas. A lithium foil was used as the anode, 1 M LiCF_3_SO_3_ in tetraethylene glycol dimethyl ether (TEGDME) as the electrolyte, glass fiber filter paper as separator, carbon-paper-supported catalysts as the cathode electrode and two piece of nickel foam (1 mm thick) as the cathode current collector^43^,^44^. The oxygen electrodes were prepared by mixing the as-prepared CNL catalysts, XC-72 carbon, 5 wt.% polyvinylidene fluoride (PVDF) and solvent N-methyl-2-pyrrolidone (NMP), and the mass ratio of CNL catalyst: XC-72 carbon: 5 wt.% PVDF is 1:2:3. The uniform slurry was coated onto the carbon-paper by a brush and dried in vacuum at 80 °C for 12 hours. The ready-made Li-O_2_ battery were transferred into a sealed bottle which was filled with pure oxygen and linked with the multichannel battery testing system (LAND CT 2001A). Galvanostatic discharge of the battery were studied in a potential range of 4.5–2.0 V. The first discharge cycle performances were conducted at the current density of 50 mA g^−1^. The following cycle performances were conducted at the current density of 250 mA g^−1^, and the capacity was limited to 500 mAh g^−1^. It should be noted that the current density and capacity were all calculated based on the mass of catalyst loaded on the cathode.

## Additional Information

**How to cite this article**: Wu, Y. *et al*. Electrocatalytic performances of g-C_3_N_4_-LaNiO_3_ composite as bi-functional catalysts for lithium-oxygen batteries. *Sci. Rep*. **6**, 24314; doi: 10.1038/srep24314 (2016).

## Figures and Tables

**Table 1:** Koutecky-Levich reasults of XC-72 Carbon, LaNiO_3_ and x wt.% CNL (x = 0, 5, 10, 20 and 30) catalysts.

Sample	Slope	*n*	Intercept
XC-72	4.98294	2.006847	0.12019
g-C_3_N_4_	4.08943	2.445329	0.11453
LaNiO_3_	3.82595	2.61373	0.05988
30 wt.% CNL	3.29416	3.035675	0.06098
5 wt.% CNL	3.26957	3.058506	0.04855
20 wt.% CNL	2.86083	3.495489	0.03153
10 wt.% CNL	2.58987	3.861198	0.02213
